# Label-free mid-infrared photothermal live-cell imaging beyond video rate

**DOI:** 10.1038/s41377-023-01214-2

**Published:** 2023-07-19

**Authors:** Genki Ishigane, Keiichiro Toda, Miu Tamamitsu, Hiroyuki Shimada, Venkata Ramaiah Badarla, Takuro Ideguchi

**Affiliations:** 1 Department of Physics, The University of Tokyo, Tokyo, Japan; 2 Institute for Photon Science and Technology, The University of Tokyo, Tokyo, Japan

**Keywords:** Mid-infrared photonics, Phase-contrast microscopy

## Abstract

Advancement in mid-infrared (MIR) technology has led to promising biomedical applications of MIR spectroscopy, such as liquid biopsy or breath diagnosis. On the contrary, MIR microscopy has been rarely used for live biological samples in an aqueous environment due to the lack of spatial resolution and the large water absorption background. Recently, mid-infrared photothermal (MIP) imaging has proven to be applicable to 2D and 3D single-cell imaging with high spatial resolution inherited from visible light. However, the maximum measurement rate has been limited to several frames s^−1^, limiting its range of use. Here, we develop a significantly improved wide-field MIP quantitative phase microscope with two orders-of-magnitude higher signal-to-noise ratio than previous MIP imaging techniques and demonstrate live-cell imaging beyond video rate. We first derive optimal system design by numerically simulating thermal conduction following the photothermal effect. Then, we develop the designed system with a homemade nanosecond MIR optical parametric oscillator and a high full-well-capacity image sensor. Our high-speed and high-spatial-resolution MIR microscope has great potential to become a new tool for life science, in particular for live-cell analysis.

## Abstract (web summary)

We develop an ultra-sensitive mid-infrared photothermal microscope, enabling molecular vibrational live-cell imaging beyond video rate.

## Graphical abstract

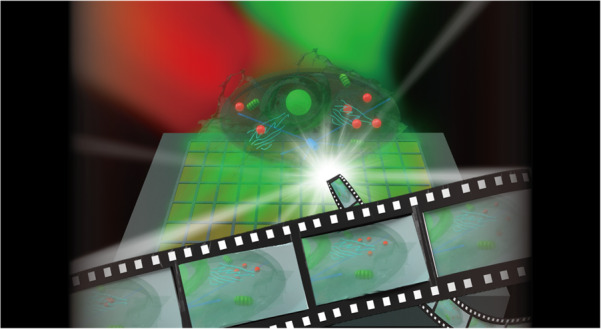

## Introduction

Vibrational imaging such as Raman scattering and mid-infrared (MIR) absorption imaging has attracted attention in life science^1,2^, e.g., in the field of single-cell biology, because its label-free capability can solve the problems associated with fluorescence imaging, such as cell damage or death due to cytotoxicity, difficulty in continuous and quantitative measurements due to photobleaching, and undesired functional modification of the labeled intracellular biomolecules^3,4^. Most single-cell vibrational imaging techniques exploit Raman scattering, and the state-of-the-art coherent Raman scattering (CRS) microscopes have achieved high-speed imaging at video rates^5,6^. These high-speed CRS imaging systems have made significant impacts in the field of vibrational imaging and triggered drastic expansion of the related research, including instrumental developments^7–11^ and biological applications^12,13^, particularly live-cell analysis. On the other hand, MIR absorption imaging is rarely used for detailed observation of single cells because of the low spatial resolution of 2–10 μm restricted by the diffraction limit of MIR light and the strong background absorption by the water surrounding the cells. However, MIR absorption imaging has great potential for life science due to the large absorption cross-section of the MIR absorption process (~10^8^ times higher than that of Raman scattering) with low photo-damage to biomolecules. CRS imaging, in contrast, exploits tightly focused ultrashort laser pulses to trigger nonlinear optical effects to perform sensitive measurements, causing undesired multiphoton electronic transitions that can cause deleterious effects to live cells^14^. MIR absorption avoids this problem because it is a single-photon linear absorption process with low photon energy. In addition, imaging with MIR light holds a possibility of obtaining information that Raman spectra have not revealed until now. MIR spectra provide rich information on biomolecules that dominantly exist in a cell, such as proteins and water, more specifically, e.g., the secondary structure of proteins via the amide band^15–17^ and the state of water molecules via the OH band^18^.

MIR photothermal (MIP) imaging is an emerging technique that has been studied in the last several years^19–32^, which can solve the above-mentioned problems and enables single-cell MIR absorption imaging. In this technique, MIR molecular absorption induces local heating in the sample, and the resulting change in refractive index is detected as changes in optical parameters such as phase^22–24,26^, reflectance^21,25,29^, or scattering intensity^19,20,27^ of visible probe light. Hence, one can obtain information on the spatial distribution of MIR absorption with a sub-μm resolution. Moreover, as long as the MIR light reaches the cell, it is possible to capture the intracellular change in refractive index due to the transparency of visible light in biological samples, even if the MIR light is subsequently absorbed by the water behind the cell.

However, the performance, particularly the frame rate, of current MIP imaging systems has not yet reached the level of the state-of-the-art CRS imaging systems. MIP imaging techniques can be classified into “point-scanning” or “wide-field” configurations. The pioneering work on single-cell imaging was demonstrated based on the point-scanning configuration, in which MIR and visible light emitted from a pulsed quantum cascade laser (QCL) and a CW laser diode, respectively, were coaxially focused on a sample. In this configuration, images were taken by scanning the sample stage, and the maximum frame rate was limited to ~0.1 Hz for taking 100 pixels × 100 pixels due to the low scanning speed of the stage and low detection efficiency of the photothermal signals. Wide-field configurations have solved this problem^21–27,29^, in which the entire field of view (FOV) is irradiated with MIR and visible light, and the wide-field photothermal signals in the FOV are detected at once with a CMOS image sensor. Since the maximum image-acquisition rate is determined as half the frame rate of the image sensor, molecular vibrational imaging beyond video rate can be realized if a high SNR is achieved. However, the image-acquisition rate in cell measurement with sub-μm spatial resolution remains in the range of 0.1 to 2 Hz^21,23,26,27^ for the state-of-the-art wide-field systems because of their low SNR due to the following reasons: (1) the low photothermal signal owing to the decrease in MIR fluence caused by wide-field illumination and the signal saturation caused by thermal diffusion, and (2) the low detection sensitivity of wide-field microscopes limited by optical shot noise due to the use of CMOS image sensors with low full-well capacity.

In this work, we develop a MIP imaging system with a high-intensity MIR nanosecond optical parametric oscillator (OPO) and highly sensitive quantitative phase imaging (QPI)^33^ using a high full-well-capacity CMOS image sensor, with which we, for the first time, realize live-cell MIP imaging beyond video rate. We employ QPI for visible detection because it enables quantitative measures of the induced phase shift. First, we perform thermal conduction simulations to derive the optimal pulse duration and repetition rate of MIR and visible light for wide-field MIP imaging: <10 ns and ~1 kHz, respectively. Then, we develop a wavelength-tunable MIR nanosecond OPO with a periodically poled lithium niobate (PPLN) crystal that meets these requirements with ~10-μJ pulses (~100-times higher pulse energy compared to that of a QCL in our previous study^24^) in the wavenumber region of 2600–3450 cm^−1^. Next, we employ a high-full-well capacity CMOS image sensor for quantitative phase measurements, capturing ~100-times more photons than conventional image sensors. The SNR of our system is evaluated to be ~210-times higher than that of the previous work^23^ with the state-of-the-art wide-field MIP-QPI system (see Discussion for details). Note that we assume a situation where compared systems measure the same molecular vibration at the same wavenumber for evaluating their SNRs. With the developed system, we perform MIP imaging of live COS7 cells in the 2925-cm^−1^ band with a high SNR of 89 at a record rate of 50 fps. The high-SNR and high-speed capabilities of our microscope are expected to be beneficial in video-rate observation of intracellular dynamics and for high-speed broadband MIR spectral image acquisition over several hundred cm^−1^ in less than 1 s.

## Results

### Derivation of the optimal pulse duration and repetition rate of MIR and visible light by thermal conduction simulations

We consider the optimal pulse duration and repetition rate of MIR and visible light for wide-field MIP imaging by exploiting thermal conduction simulations (see Methods for details). Thermal diffusion causes degradation of spatial resolution and saturation/decay of the amount of signals in the MIP imaging. The change in optical phase-delay of visible light due to the local temperature rise, which we call the MIP phase change, is expressed as

1 $$\Delta \theta (x,y,t) \sim \frac{2\pi }{\lambda }\frac{{{\rm{d}}n}}{{{\rm{d}}T}}\int \Delta T(x,y,z,t){{\rm{d}}z}$$

where *x*, *y*, *z* denote spatial coordinates, *t* the time, *λ* the wavelength of visible light, *dn*/*dT* the thermo-optic coefficient of the sample, and Δ*T* the local temperature change. The temporal evolution of the MIP phase change under various MIR excitation conditions in an aqueous environment is calculated by solving the 3D heat conduction equation,

2 $$\frac{\partial \Delta T(x,y,z,t)}{\partial t}=\left(\frac{{\partial }^{2}}{\partial {x}^{2}}+\frac{{\partial }^{2}}{\partial {y}^{2}}+\frac{{\partial }^{2}}{\partial {z}^{2}}\right)\nu \Delta T(x,y,z)+\frac{I(x,y,z,t)\alpha (x,y,z)}{\rho {c}_{{\rm{p}}}}$$

where *ν* denotes the thermal diffusivity, *I* the pulse fluence per unit time, *α* the absorbance, *ρ* the density, *c*_p_ the specific heat capacity.

**Fig. 1:**
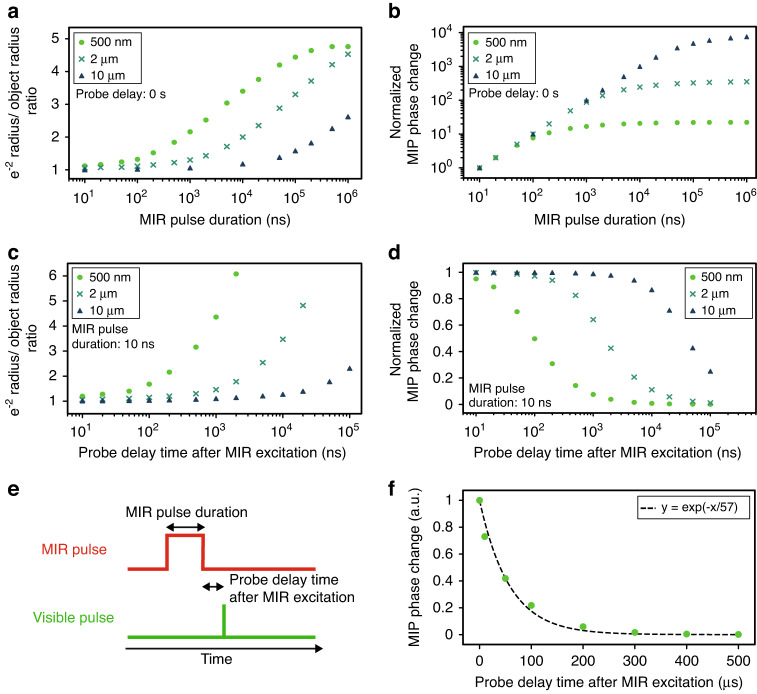
Derivation of optimal pulse duration and repetition rate of MIR and visible light by thermal conduction simulations. **a** Degradation of the spatial resolution in MIP imaging originating from thermal diffusion depending on the MIR pulse duration. The horizontal axis is the MIR pulse duration, and the vertical axis is the ratio of the e^−2^ radius in the MIP phase change image and the radius of the original spheres. **b** Saturation of the MIP phase change depending on the MIR pulse duration. MIP phase changes at the centers of the spheres are plotted against MIR pulse durations, which are normalized by that with the MIR pulse duration of 10 ns. The probe delay time after MIR excitation is 0 s for (**a**) and (**b**). **c** Degradation of the spatial resolution in MIP imaging originating from thermal diffusion depending on the probe delay time after MIR excitation. **d** Decay of the MIP phase change depending on the probe delay time after MIR excitation. The MIP phase changes are normalized by that with the probe delay of 0 s. MIR pulse duration is set to 10 ns for (**c**) and (**d**). **e** Pulse duration and timing chart of the MIR and visible pulses. **f** Temporal decay of the MIP phase change for water (10 µm thickness) sandwiched between two CaF_2_ substrates. The vertical axis shows the MIP phase change at the center of the heated spot. The pulse duration of the MIR light is assumed to be much shorter than the thermal decay time. The spatial distribution of the MIP phase change is assumed as a gaussian function (FWHM = 91 µm) along the *x*- and *y*-axes, determined by the intensity profile of the MIR spot, and an exponential function along the *z*-axis that decays after 16 µm, which is derived from the Lambert-Beer law

To derive the optimal pulse duration of MIR light, we calculate the spread of the spatial profile (Fig. 1a) and the phase change (Fig. 1b) in the MIP phase change image with respect to the pulse duration of MIR light. We assume the initial heat spots (target objects) are spheres with a diameter of 500 nm, 2 µm, and 10 µm in aqueous environments. They have the same thermal diffusivity as water and are continuously heated by the MIR pulse with constant peak power, which represents a situation using a QCL, illustrating the disadvantages of using long pulses. We assume that the visible probe pulse is sufficiently shorter than the MIR pulse and illuminated at the end of the MIR pulse. Figure 1e shows the timing chart of the MIR and visible pulses. The results show that a longer pulse builds up the MIP phase change, but too much elongation leads to degradation of the spatial resolution and signal saturation due to thermal diffusion, particularly for small objects with a large surface/volume ratio. The saturation time is proportional to the square of its radius. For example, when observing the 500-nm object, a 100-ns MIR pulse deteriorates the spatial resolution by a factor of 1.3 (Fig. 1a). Some works exploit a CW MIR light source^22,25^ for continuous heating. However, in such cases, the thermal spread is ~4.8-times larger than the actual size of the target, and the MIP phase change of the 500 nm object remains constant after 100 ns. In addition, it causes a lack of quantitative capability due to the discrepancy in saturation time dependent on the object size (Fig. 1b). To better illustrate a situation employing a ns-OPO, we also conduct the same simulation for constant pulse energy (see Supplementary Note 2). These results demonstrate that MIR pulses of ~10 ns or shorter are desirable for quantitative imaging by confining the generated heat within a near-diffraction-limited spot of visible light, promoting the use of a ns-OPO with a high pulse energy instead of a long-pulsed or a CW QCL.

Next, to derive the optimal pulse duration and delay of the visible light, we calculate the spread of the spatial profile (Fig. 1c) and the phase change (Fig. 1d) in the MIP phase change image with respect to the delay of the visible probe light from the end of the MIR excitation. In this calculation, the MIR pulse duration is 10 ns, and the visible probe pulse is sufficiently shorter than the MIR pulse. The results show that the probe delays longer than 10 ns cause degrading the spatial resolution (Fig. 1c) and decreasing the phase change (Fig. 1d) of the MIP phase change image. For example, when observing the 500-nm object with a delay of 100 ns, the radius of the MIP phase change becomes 1.9-times larger than the object, and the MIP phase change decays to 50% of that with a delay of 0 s. This simulation shows that it is desirable for visible probe pulses to have a similar or shorter pulse duration than MIR pulses, i.e., <10 ns, with the delay time shorter than the pulse duration. This result indicates that visible light sources that pose difficulties in ensuring pulse energy, such as LEDs, are not the optimal probes for the MIP effect.

Finally, to derive the optimal pulse repetition rate, we calculate the thermal diffusion time of a heated spot with an FWHM diameter of 91 µm in an aqueous environment which reflects the condition of our following experiment (Fig. 1f). Note that, when measuring cells, the thermal diffusion time over the entire FOV does not depend on the size of the target objects but on the spot size of MIR light due to water absorption. To avoid potential thermal damage to samples due to a thermal pile-up, the induced photothermal heat should be diffused off when the next MIR pulse arrives at the sample. The result shows that this condition is sufficiently achieved at 1 ms after the arrival of the first MIR pulse. Hence, it is desirable for wide-field single-cell imaging to exploit a pulse repetition rate of ~1 kHz.

Supplementary Note 1 compiles the parameters of existing wide-field systems, which allows for comparative analyses. For example, the MIR pulse energies in Table S1 can be used for estimating MIP phase change induced by different MIR light sources, such as a pulsed OPO, a pulsed QCL, and a CW QCL, based on the knowledge provided by the simulation results shown in Fig. 1b (see Supplementary Note 1 for details). Table S1 shows that the previously demonstrated systems do not satisfy the optimal condition determined by our simulations.

### Highly sensitive MIP-QPI system

**Fig. 2:**
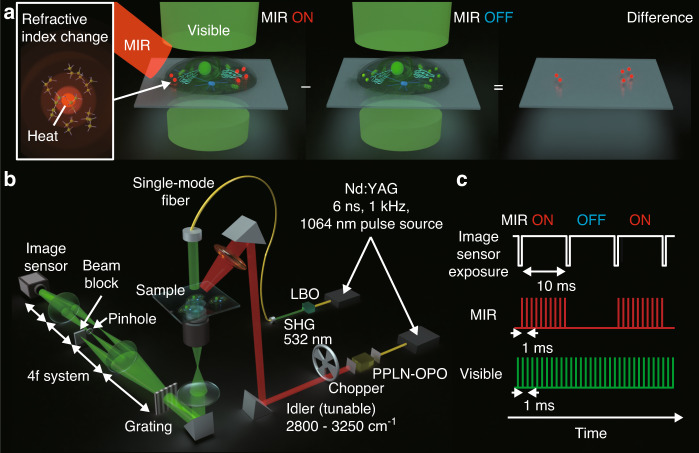
Principle and schematic of MIP-QPI. **a** Principle of MIP-QPI. **b** Optical system of high-SNR MIP-QPI. **c** Timing chart of MIR and visible pulses. MIR mid-infrared, LBO LiB_3_O_5_, SHG second harmonic generation, PPLN periodically poled lithium niobate, OPO optical parametric oscillator

The principle and the schematic of our newly developed MIP-QPI are shown in Fig. 2. MIR light with a narrow spectral width at a certain wavenumber is irradiated widely over the sample. Resonant molecules absorb the MIR light and are excited to their vibrational states. The molecular vibrations relax by transferring their energy to the surrounding medium in the form of heat, causing thermal expansion and thus a change in local density. The resulting change in refractive index in the vicinity of the target molecules (i.e., MIP effect) is captured as a change in optical phase delay of the transmitted visible light in the QPI system^33^. As shown in Fig. 2a, a MIP image is generated by taking the difference between the phase images captured in the MIR-ON and -OFF states. Amongst the available detection methods of the MIP effect, QPI is the optimal method for the quantitative measurement of intracellular molecular distributions. For example, phase-contrast microscopy^34^ suffers from image artifacts such as halos, while dark-field^35^ and interferometric scattering (iSCAT)^36^ microscopes sacrifice a part of the spatial-frequency information. QPI does not have those drawbacks and provides quantitative MIP images. In addition, detailed morphology with dry-mass information can also be obtained from the quantitative phase image in the MIR-OFF state. Therefore, one can make a correlation analysis between the spatial distribution of target molecules and cell organelles.

Figure 2b shows the schematic of our MIP-QPI system developed in this study. Two Nd:YAG Q-switched lasers (1064-nm wavelength, 1-kHz repetition rate, 6-ns pulse duration) (NL204, Ekspla) are used to generate visible and MIR pulses via nonlinear wavelength conversions. The visible light pulses (532-nm wavelength, 1-kHz repetition rate, 5-ns pulse duration) are provided by second harmonic generation (SHG) with a 15-mm-long LBO crystal. The MIR light pulses (2800–3250 cm^−1^ wavenumber tunable, 1-kHz repetition rate, 9-ns pulse duration, ~10-µJ pulse energy) are obtained as idler pulses of a homemade high-intensity nanosecond OPO with a fan-out PPLN crystal^37^ (HC Photonics Corporation). The visible pulse is electronically synchronized with the MIR pulse using a function generator. In our current system, we set a delay of ~100 ns between the MIR and visible pulses, which is longer than the theoretical optimum because there is a timing jitter up to 50 ns between the pump and probe pulses. The 100-ns delay guarantees the visible probe pulses come after the MIR pump pulses under this amount of jitter. Note that we can suppress the jitter down to ~1 ns, which is specified in a product specification sheet of our lasers, by appropriate synchronization. The MIR light is intensity-modulated by a mechanical chopper at 50 Hz synchronized with the image sensor frames such that the sensor alternately acquires MIR-ON and -OFF frames (Fig. 2c). The MIR light pulses are loosely focused onto the sample with a spot size of ~80 µm × 80 µm with a ZnSe lens. The visible light pulses from the single-mode fiber are collimated and irradiated onto the sample with a peak fluence of ~30 pJ µm^−2^ (~400 nJ over 110 µm × 110 µm). Common-path off-axis digital holography is used as QPI^38^. The light transmitted through the sample is replicated by a diffraction grating, and the zeroth-order diffraction light is low-pass filtered with a pinhole placed in the Fourier plane, thus, converted to a quasi-plane wave that acts as the reference light. The first-order diffraction light is used as the object light, which contains information on the optical phase delay induced by the sample. Interference fringes between the two lights are captured as an off-axis hologram with the high-full-well-capacity image sensor (Q-2HFW, Adimec Advanced Image Systems) after relay lenses in 4f configuration, from which the phase image is numerically reconstructed. The experimentally evaluated spatial resolution of QPI is 440 nm, determined by the NA of the objective lens (LUCPLFLN40X, Olympus). Our system is resistant to speckle noises due to the use of a temporally low-coherent ns-visible light with a bandwidth of a few nm in the temporal differential measurement.

### High-intensity nanosecond MIR light source

**Fig. 3:**
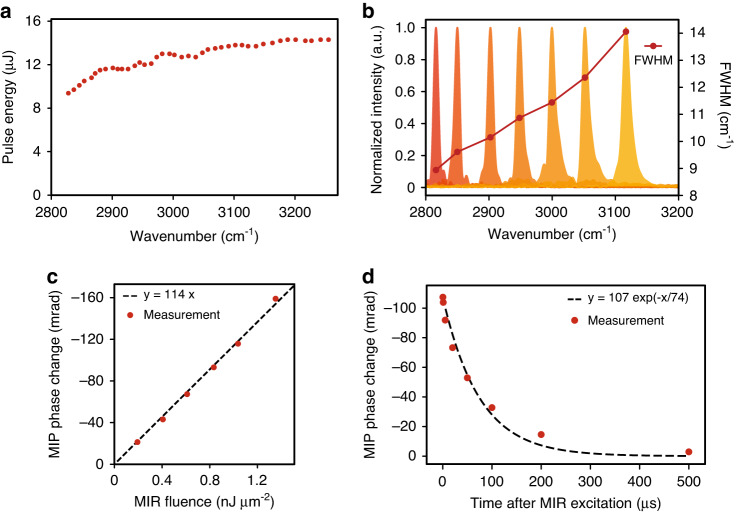
Basic performances of MIR light source (ns-OPO). **a** MIR pulse energy in the range of 2800–3250 cm^−1^. **b** Spectra of MIR light measured with a homemade FTIR spectrometer. The red points indicate the FWHM of each spectrum. **c** Linearity of MIP phase change with respect to MIR fluence for a water sample excited at 2918 cm^−1^. **d** Temporal decay of MIP phase change for a water sample with an excitation area of 69 µm × 69 µm. FWHM full width at half maximum

We describe the performance of our homemade ns-PPLN-OPO (see Supplementary Note 3 for details). The crystal is a 50-mm-long fan-out PPLN with a poling period varying from 27.5 to 31.6 µm stabilized at 40 °C. The pulse energy of the pump light from the Nd:YAG Q-switched laser is ~100 µJ. The OPO cavity is resonant with the NIR signal pulses (5950–6800 cm^−1^ tunable), and only the MIR idler pulses (2600–3450 cm^−1^ tunable) are extracted with a long-pass filter after the cavity. Figure 3a shows the relationship between the MIR wavenumber and the idler pulse energy, which is ~10 µJ between 2800 and 3250 cm^−1^. Figure 3b shows the spectrum of MIR light measured by a homemade FTIR spectrometer. The FWHM of the spectrum is ~10 cm^−1^ at each MIR wavenumber, which determines the spectral resolution and is sufficient to resolve absorption peaks of CH_3_ and CH_2_ stretching modes (the modes are 20 ~ 30 cm^−1^ apart from each other)^39^, which are the major signatures of analyzing biological samples in this wavenumber range. Figure 3c shows how the MIP phase change of water changes with respect to the MIR pulse energy under the condition of the MIR wavenumber of 2918 cm^−1^, the spot size of 69 µm × 69 µm, and the pulse energy ranging from 1.3 to 8.9 µJ. The values of the MIP phase change in the graph are averages of 20 pixels × 20 pixels around the center of the MIR spot. The results show that the MIP phase change increases linearly with the MIR pulse energy over the entire range, which indicates that our OPO yields a ~100-times larger MIP phase change compared to the previous work with a QCL^24^. Finally, the temporal decay of the MIP phase change in water is measured by scanning the time delay between the MIR and visible light pulses (Fig. 3d). One can see that the MIP phase change decays to 1/e at 74 µs and to 3/100 at 500 µs, which is consistent with the results derived from the thermal conduction equation (1/e at 57 µs). Since it is desirable to observe live cells under a similar or larger MIR illumination spot size, a repetition rate of 1 kHz is low enough to avoid potential sample damage induced by a thermal pile-up.

### High-precision QPI system

We discuss noise reduction in phase measurement with QPI by employing a high full-well-capacity image sensor and a high-intensity ns visible light. If the system is mechanically stable enough, the temporal phase noise in QPI can be dominated by optical shot noise. Thus, the precision becomes higher when more light enters the image sensor. The full-well capacity of the image sensor used in our system is 2 Me^−^ pixel^−1^ (Q-2HFW, Adimec), which is 200 times larger than that of a conventional CMOS image sensor (10 ke^−^ pixel^−1^, e.g., acA2440–75um, Basler). We perform the following evaluations of the noise reduction.

**Fig. 4:**
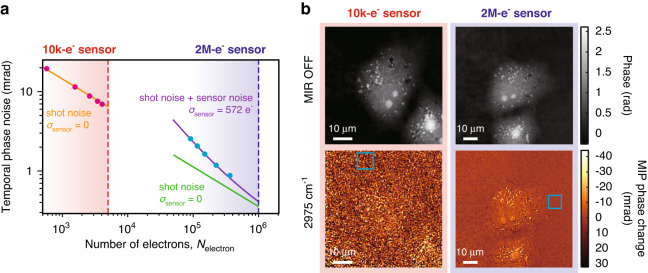
Noise reduction in phase measurement with QPI. **a** Dependence of temporal phase noise on the number of electrons generated in image sensors. Red and blue dots are measured data with a conventional 10k-e^−^ image sensor (acA2440–75um, Basler) on the left side and a high full-well-capacity 2M-e^−^ image sensor (Q-2HFW, Adimec) on the right side, respectively. Orange and green lines are theoretically estimated phase noise from Eq. 3 in “Methods” with sensor noise *σ*_sensor_ = 0, and the purple line is that with *σ*_sensor_ = 572 e^−^ (detailed parameters are written in Methods). **b** Comparison between MIP images taken with the two sensors: phase images (top) and MIP phase change images (bottom) of live COS7 cells taken with 10k-e^−^ (left) and 2M-e^−^ (right) sensors. The number of electrons per pixel^,^ *N*_electron_, is 2.82 × 10^3^ e^−^ and 2.34 × 10^5^ e^−^ with 10k-e^−^ and 2M-e^−^ sensors, respectively. MIR light at a wavenumber of 2975 cm^−1^ is irradiated on a spot size of 80 μm × 80 μm with a fluence of 1.1 nJ µm^−2^

We examine the dependence of temporal phase noise on the average number of electrons contributing to the reconstruction of the phase images (=*N*_electron_) per sensor’s pixel (Fig. 4a), that is, the average number of electrons in the holograms (see Supplementary Note 4 for the calculation method). The maximum value of *N*_electron_ is determined as half the full-well capacity of the image sensor. Note that *N*_electron_ equals the number of incident photons multiplied by the quantum efficiency of the image sensor. We record 100 holograms without a sample and calculate the differences in phase images between adjacent frames. Then, the temporal standard deviation (STD) of the 50 differential images is calculated at each pixel, and the average value of 80 pixels × 80 pixels in the temporal STD map is evaluated as the temporal phase noise. The number of electrons per pixel is estimated from the sensor’s digital output value and the full-well capacity. The data points on the left side of the graph are the measurement results using the conventional 10k-e^−^ image sensor, which is in good agreement with the theoretically estimated phase noise limited by optical shot noise (orange line)^40^ (see Eq. 3 in “Methods” for details). This indicates that the phase noise can be reduced by detecting more light. The data points on the right are the results using the high-full-well-capacity 2M-e^−^ image sensor. One can see that the maximum number of detected electrons is ~100-times larger than that measured with the 10k-e^−^ image sensor, resulting in a reduction of the phase noise by a factor of 7.9 (corresponding to 0.9 mrad with *N*_electron_ = 3.6 × 10^5^ e^−^). However, this phase noise is larger than that determined by optical shot noise and is in good agreement with the estimated value (purple line) that includes the effect of sensor noise (*σ*_sensor_ = 572 e^−^), measured by turning off the laser. It is expected that near optical shot-noise-limited measurement (0.4 mrad of the phase noise) is feasible when the sensor’s full-well capacity is used to the maximum extent (*N*_electron_ = 1 × 10^6^ e^−^).

Next, we compare the SNR of single-frame MIP phase change images of live cells measured with the two image sensors. Figure 4b shows results for the observation of COS7 cells exploiting MIR light with a wavenumber of 2975 cm^−1^, a spot size of 80 μm × 80 μm, and pulse energy of 7.1 µJ. The background MIP phase change image of water without cells is subtracted to make the intracellular structures more visible. The spatial STD of 20 pixels × 20 pixels inside the blue box is defined as the phase noise. Note that Fig. 4b has ~√2-times larger noise than the temporal phase noise in Fig. 4a due to the background subtraction process. In the case of the 10k-e^−^ sensor, the MIP phase change is buried in the phase noise, whereas in the case of the 2M-e^−^ sensor, intracellular structures such as nucleoli and lipid droplets are clearly seen. The phase noise is 12.2 mrad for the former sensor and 1.6 mrad for the latter (8.6 mrad and 1.1 mrad without water background subtraction, respectively). We verify that the high full-well capacity sensor provides ~7.6-times reduction in phase noise with 85-times higher power of visible light for the live-cell imaging.

### Video-rate MIP imaging of a single live cell

**Fig. 5:**
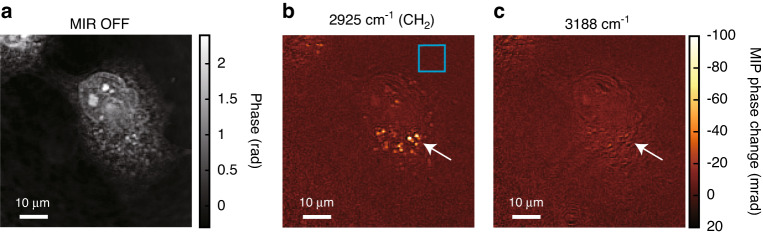
Video-rate MIP imaging of a live COS7 cell. **a** A phase image. **b**, **c** Single-frame MIP phase change images excited at 2925 and 3188 cm^−1^ with a spot size of 87 µm × 87 µm. The images are taken at a rate of 50 Hz (20 ms measurement time per image)

We demonstrate MIP imaging of a live COS7 cell at 50 fps. Figure 5a is a phase image measured in MIR-OFF state, and 5b and c are MIP phase change images with water background subtraction, excited at 2925, and 3188 cm^−1^ MIR wavenumbers, respectively. Note that they are all single-frame images without averaging. The MIR pulse energy at the sample plane is ~6.5 μJ with a spot size of 87 μm × 87 μm. The image at 2925 cm^−1^ contains strong signals mainly from CH_2_ bonds of lipid droplets indicated by the white arrow, while the image at 3188 cm^−1^ shows signals that hardly reflect intracellular structures. Thus, different contrasts are observed at different MIR wavenumbers at an unprecedentedly high measurement rate of 50 Hz (20 ms measurement time per image). The phase noise, i.e., the spatial STD of 20 pixels × 20 pixels inside the blue box in Fig. 5b, is evaluated as 1.6 mrad with water background subtraction by following the procedure used in Fig. 4. Since the signal from the lipid droplets is ~100 mrad, the SNR is 63 (89 without water background subtraction). Hence, high-SNR live-cell MIP imaging beyond video rate is achieved for the first time.

### Video-rate MIP imaging of sub-second cellular dynamics

To exemplify the capabilities of high-speed MIP imaging for more practical cellular dynamics, we observe cellular dynamics on a sub-second scale, specifically, the transfer of water molecules through aquaporins—membrane proteins that function as water-selective channels and control the intracellular water content^41^. Its study has been challenged by the difficulty in accurately measuring the flux of water into and out of living cells. In this experiment, we assess the transient changes in intracellular MIP effect originating from H_2_O when D_2_O and H_2_O are rapidly interchanged, analogous to previous CRS studies^42^. As noted in the introduction, MIP imaging is expected to be an ideal method for this application owing to the large MIR absorption cross-section of a water molecule.

**Fig. 6:**
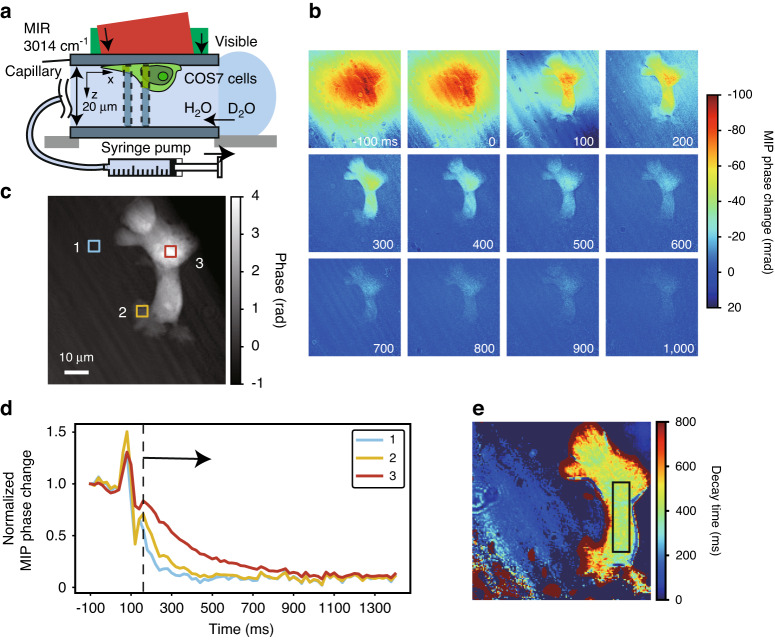
Video-rate MIP imaging of an H_2_O/D_2_O exchange in a live COS7 cell through aquaporins. **a** A schematic of the measurement platform, wherein H_2_O-based PBS filled in a capillary is exchanged for D_2_O-based PBS via a syringe. A percentage of the cell’s space occupation along the *z*-axis of the capillary varies depending on the detection regions, denoted by dot squares. **b** Series of MIP images at 3014 cm^−1^ recorded at 50 fps. **c** A phase image. **d** The average temporal decay within the square regions in Fig. 6c, normalized by the MIP phase change at −100 ms. **e** A distribution of intracellular decay times (*τ* in Eq. 4) derived from fitting temporal decays. The fitting range is chosen on the longer side of the dotted line shown in Fig. 6d in order to circumvent the signal fluctuation around 100 ms

Figure 6a illustrates a schematic diagram of the measurement platform, wherein a capillary filled with H_2_O-based phosphate-buffered saline (PBS) is exchanged for D_2_O-based PBS via a syringe pump within a time frame shorter than the sensor’s frame interval. We present a series of images depicting temporal evolutions in MIP phase change recorded at 50 fps (Fig. 6b). The measured movie can be seen in Supplementary Video 1. Between −100 and 0 ms, an H_2_O-induced MIP phase change reflecting the MIR spot is visibly apparent. From 0 to 300 ms, the extracellular signal promptly declines upon substitution with D_2_O-based PBS, and only the intracellular MIP signal produced by H_2_O molecules remains, which eventually decays over the course of several hundred milliseconds. Figure 6c, d display the phase image of the cell in the MIR OFF state and the temporal decay of MIP phase changes at various sites indicated in Fig. 6c, respectively. The signal fluctuation around 100 ms in Fig. 6d is an artifact caused by slight agitation of the capillary during the liquid exchange process, which can be resolved through refinement of the capillary fixation. The sites exhibiting larger phases, i.e., thicker cellular sites, manifest slower decay, as the imaging was performed in the presence of both water and cells in the z-direction, resulting in different ratios of the two signal types (dot squares in Fig. 6a). In order to estimate the intracellular decay time at each site, the height distribution inferred from the phase information and the extracellular decay time are substituted for Eq. 4, described in Methods, prior to the fitting procedure. The intracellular decay time is found to be nearly constant within the central portions of the cells, with an average of 420 ms within the square region depicted in Fig. 6e. When investigating the function of aquaporins, it is important to determine the velocity of water molecules traversing the cell membrane, *P*_*d*_, because the decay time, *τ*, varies in accordance with the shape and size of the cell. In the previous study^42^, these values were obtained through separate measurements using a confocal fluorescence microscope, whereas our microscope possesses the advantage of being able to estimate the height and surface area from the morphological information acquired by QPI in the MIR OFF state (see Methods for details). Upon substituting these values into Eq. 8, *P*_*d*_ is calculated to be 6.4 × 10^−4 ^cm s^−1^, which agrees well with prior observations in HeLa cells^42^. This demonstration verifies the ability to observe intracellular phenomena on a sub-second scale by leveraging the high measurement speed of our microscope.

### Broadband MIP spectro-imaging of a single live cell

We measure spectra of a single live COS7 cell and perform multivariate analysis as one of the applications utilizing the high SNR of our system. By scanning the wavenumber of the MIR light, 40 MIP phase change images are acquired in the range of 2800 ~ 3250 cm^−1^. The MIR pulse energy at the sample plane is ~6.5 μJ with a spot size of 85 μm × 85 μm. The total acquisition time is ~10 min for 500 MIP phase change images averaged at each wavenumber, which is limited by the performance of the controlling system for spectral acquisition (see Discussion for more details). The hyperspectral data are subjected to multivariate analysis (Multivariate curve resolution, MCR)^43^ to extract characteristic components (see “Methods” for details). Three clearly interpretable components are chosen for the analysis.

**Fig. 7:**
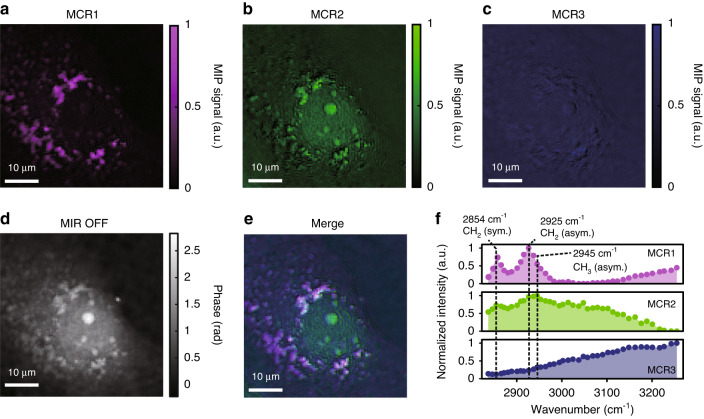
Multivariate (MCR) analysis of a MIP spectro-image of a live COS7 cell. MIP phase change images excited at 40 different wavenumbers in the range of 2800 ~ 3250 cm^−1^ are analyzed by the MCR method. **a**–**c** Images of each MCR component. **d** A phase image. **e** A merged image of the three MCR images. **f** MIR spectra of each MCR component. MCR multivariate curve resolution. sym symmetric vibrations. asym asymmetric vibrations

Figure 7a–c, d, e, and f shows spatial distributions of the three MCR components, a phase image in a MIR-OFF state, a merged image of the three MCR images, and spectra of the three MCR components, respectively. In MCR1, the MIP contrasts are localized at extranuclear lipid droplets, and two peaks at 2854 cm^−1^ and 2925 cm^−1^ corresponding to symmetric and asymmetric stretching vibrations of CH_2_ bonds appear in the spectrum, indicating that MCR1 mainly consists of lipids. In MCR2, the MIP contrasts are localized at the nucleus and nucleolus, and the spectrum is heavily influenced by the peak attributed to CH_3_, indicating that it is a component with equal contributions of CH_2_ and CH_3_ bonds, which can be mainly attributed to proteins. In MCR3, uniformly distributed contrasts outside the cell can be recognized, and its spectrum resembles that of OH bonds, which have an absorption peak around 3400 cm^−1^ and monotonically increasing absorption towards higher wavenumbers in the observed wavenumber region^18^, indicating that water is the main contributor. Thus, based on the vibrational modes of CH_2_, CH_3_, and OH, we are able to separate the three basic components of the cell. The spectral shapes are slightly unnatural in the sense that the high wavenumber side of MCR1 is elevated, and the CH_3_ absorption peak of MCR2 is ambiguous. However, the problem does not occur in a similar measurement where the medium is replaced with D_2_O-based PBS to eliminate the effect of absorption by OH bonds (see Supplementary Note 5 and Fig. S4). Hence, it can be considered that the spectra are slightly distorted during MCR analysis due to the stronger absorption of water compared to other components, which is discussed more in the discussion section.

The MCR1 component at 2925 cm^−1^ (CH_2_ peak) induces the MIP phase changes of 40 mrad at the lipid droplets, while the MCR2 component at 2945 cm^−1^ (CH_3_ peak) induces only 14 mrad changes in the nucleolus, and 3 mrad changes in the cytoplasm, which is calculated with the procedure shown in Methods. Since the phase noise of our QPI is ~1.1 mrad, even smaller phase changes can be detected without averaging. The estimated maximum temperature rise of this measurement is ~8 K for a lipid droplet (a sphere with a diameter of 3 µm) and ~2 K for a nucleolus (a sphere with a diameter of 5 µm), which quickly decays within ~2 and ~7 µs, respectively. These amounts of transient temperature rise have been proven to be safe for live cells^44^.

## Discussions

We make an SNR comparison between our system and the previous state-of-the-art wide-field MIP imaging system based on QPI^23^. Here, we assume a situation where the compared systems measure the same molecular vibration at the same wavenumber for evaluating their SNRs. The MIR pulse energy of our system is 6.5 μJ, while that of the previous work is 110 nJ, enabling ~59-times higher MIP phase change generation with our system. For visible imaging, the sensor’s full-well capacity of our system is 2 Me^−^, while that of the previous work is 30 ke^−^. Considering the sensor and shot noises, ~7.1-times higher SNR is achievable with our system. In the current system, however, we only use *N*_electron_ = 360 ke^−^ due to limitation of the photon budget that can be coupled to the single-mode optical fiber without damage, which gives ~3.5-times higher SNR if we assume the previous work fully uses the sensor’s capacity (*N*_electron_ = 15 ke^−^). In total, our system can provide a higher SNR than the previous work by up to ~420 times if using the full capability of the system or by ~210 times with the current demonstration with the limited visible photon budget. We note that this comparison is based upon the same conditions with the pulse repetition rate of 1 kHz and the sensor’s frame rate of 100 Hz.

Next, we discuss the spectral distortion in the MCR analysis, which is observed on the higher wavenumber side. This could be due to the low SNR around 3200 cm^−1^ due to the large water absorption. This issue could be mitigated by omitting the spectral range where absorption is primarily due to the water. In our proof-of-concept demonstration, however, we include this range to show the broad spectral coverage of our system. Another potential cause of the spectral distortion would be the imperfection of intensity calibration of MIR light. If the MIR pulse energy of each wavenumber is not accurately calibrated, a spectral distortion could occur in the MCR analysis because potentially weak signals from biomolecules can be overlapped with the larger water absorption background. It could be resolved by accurate calibration by monitoring the MIR pulse energy for every measurement.

There is room for further technical improvements in our microscope. The first is to broaden the tunable spectral range of the MIR nanosecond OPO, covering the molecular fingerprint region by using other nonlinear crystals such as AGS^45^, BGSe^46^, or OP-GaP. This could enable ultra-broadband MIR spectroscopic imaging in the range of 600–3700 cm^−1^^46^. The second is to speed up spectral imaging. In the experiment of Fig. 7, the measurement speed is limited by the data writing speed of the acquired images to a storage medium. Therefore, the overall acquisition time is unchanged even when averaging over 500 images with the current system. This issue can be resolved by direct storage of image data in the camera, which enables high-speed data acquisition of 50 spectral images within one second. The third is to further improve the detection sensitivity and imaging speed. In this experiment, the sensor’s full-well capacity (2 Me^−^) and maximum frame rate (500 fps) are not used to the full extent due to insufficient light intensity. This is because the intensity of irradiated visible light is limited by the damage threshold of the single-mode fiber. This can be solved by using a large-core single-mode fiber often used for high-power lasers. The intensity of visible light can be increased by a factor of ~20, which enables ultrafast MIP imaging at a maximum imaging rate of 250 fps (limited by the frame rate of our image sensor) with an improved SNR up to 250. It could also be possible to further increase the detection sensitivity by combining our system with a highly sensitive QPI using a wavefront shaping technique (ADRIFT-QPI)^26^. The fourth is an extension to high-speed 3D imaging. QPI can be extended to optical diffraction tomography (ODT), in which 3D refractive index distributions can be obtained by imaging with, e.g., multiple illuminations at different angles. With a commercially available high-speed spatial light modulator (SLM), which can change the illumination pattern at ~500 Hz, it could be possible to perform the world’s first 3D vibrational imaging at a video rate that has not yet been achieved even with coherent Raman imaging.

Finally, we examine the potential applications that could be achieved with the current and improved systems. The observable bandwidth of the current system lies in the range of 2800–3400 cm^−1^, enabling spectroscopic imaging of CH, amide A and B bands, and OH bands. For instance, it is feasible to visualize intracellular dynamics of water through differential spectra of H_2_O and D_2_O (depicted in Fig. 6), to evaluate cellular senescence by observing the temporal variations in the CH_2_ signal^47^, and to visualize specific carbohydrates such as paramylon present in euglena gracilis^48^. However, comprehensive observation of proteins, DNA, RNA, and glucose is challenging with the current system. The use of a broadband-tunable MIR light source in the molecular fingerprint region would be advisable for extensive analysis of various intracellular molecules, such as molecular-specific mass imaging enabled by correlation analysis with quantitative phase and MIP images^49^. Other promising applications with high-speed imaging are, e.g., video-rate observations of sub-second biological phenomena such as cell signaling^50^ via amide bands (1500–1700 cm^−1^), bacterial spore germination^51^ via calcium dipicolinate (CaDPA)-dominated band (~1010 cm^−1^).

## Methods

### Preparation of biological samples

COS7 cells are cultured on a CaF_2_ substrate with a thickness of 500 μm in high glucose Dulbecco’s modified eagle medium with L-glutamine, phenol red, and HEPES (FUJIFILM Wako) supplemented with 10% fetal bovine serum (Cosmo Bio) and 1% penicillin-streptomycin-L-glutamine solution (FUJIFILM Wako) at 37 °C in 5% CO_2_, and are sandwiched with another CaF2 substrate before imaging. For live-cell imaging in D_2_O environment (Fig. S4), the medium is replaced by D_2_O-based PBS. Note that MIP imaging with glass substrates is feasible in the spectral range observed in this work.

### Thermal conduction simulations

For Fig. 1a–d, the spherically symmetric 3-D thermal conduction equation is exploited. We use a self-made program based on the Forward Time Centered Space (FTCS) method, implemented by C programming language. We assume that the thermo-optic coefficient and heat capacity of the objects are equivalent to water because potential heat sources in cells are predominantly composed of water (~80%). We also assume that the thermo-optic coefficient does not depend on temperature. The background water absorption with a profile of ~100 μm × 100 μm is not taken into account in Fig. 1a, b because it generates a temperature change profile with a much shallower gradient compared to that of the smaller target object. We do not take into account the spatial resolution of the microscope for plotting the phase changes in Fig. 1.

For Fig. 1f, Eq. 2 is used with the boundary conditions between water and CaF_2_ substrates given by

$${K}_{{\rm{water}}}{\left(\frac{\partial {T}_{{\rm{water}}}(x,y,z,t)}{\partial z}\right)}_{z={\rm{boundary}}}={K}_{{\rm{Ca}}{{\rm{F}}}_{2}}{\left(\frac{\partial {T}_{{\rm{Ca}}{{\rm{F}}}_{2}}(x,y,z,t)}{\partial z}\right)}_{z={\rm{boundary}}}$$

where *K*_water_ and ${K}_{{\rm{Ca}}{{\rm{F}}}_{2}}$ are the thermal conductivities of water and CaF_2_, respectively. In the calculations, the thermal diffusivities and thermal conductivities of water and CaF_2_ substrates are 0.146 and 2.92 µm^2^ µs^−1^, and 0.618 and 9.71 W m^−1^ K^−1^, respectively^52,53^.

### Parameters in phase sensitivity evaluation

The temporal phase noise, *σ*_phase_, can be described as,

3 $${{\rm{\sigma }}}_{\text{phase}}=2\sqrt{\frac{[{N}_{{\rm{electron}}}+{{(\sigma }_{\text{sensor}})}^{2}]{A}_{\text{aperture}}}{{v}^{2}{{(N}_{{\rm{electron}}})}^{2}{A}_{{\rm{sensor}}}}}$$

where *σ*_sensor_ denotes the sensor noise, *v* the visibility of the hologram, *A*_sensor_ and *A*_aperture_ the numbers of pixels in total and cropped areas in the spatial frequency space. The number of electrons contributing to the reconstruction of a phase image, *N*_electron_, is calculated from the image sensor output value with sensor’s parameters of full-well capacity, bit depth (2M-e^−^ sensor: 11 bit, 10k-e^−^ sensor: 16 bit), and gain (2M-e^−^ sensor: 1.73, 10k-e^−^ sensor: 1). To obtain *σ*_sensor_, a series of images are taken without light, and the temporal standard deviation of the difference images between adjacent frames is calculated, which is converted to the number of electrons. The visibility *v* is evaluated by the procedure described in Supplementary Note 4. The numbers of pixels *A*_sensor_ and *A*_aperture_ are 2,073,600 (1440 pixels × 1440 pixels) and 47,144 (π/4 × 245 pixels × 245 pixels) for the 2M-e^−^ sensor, and 1,046,529 (1023 pixels × 1023 pixels) and 31,731 (π/4 × 201 pixels × 201 pixels) for the 10k-e^−^ sensor, respectively. Note that the reduction in the number of pixels occurred in the phase reconstruction process (*A*_sensor_ → *A*_aperture_) results in a reduction of phase noise due to the spatial averaging effect.

### Procedure in H_2_O/D_2_O exchange observation in a live COS7 cell

A 20-μm-thick borosilicate glass capillary (VitroTubes 5002, VitroCom) is connected to a syringe via a PEEK tube (1/32 "OD × 0.02 "ID, Trajan) on one side. A droplet of D_2_O-based PBS is placed on the other end of the capillary, and the liquid inside is replaced by pulling on the syringe.

The function of the curve fitting of the measured temporal data shown in Fig. 6d is written as

4 $$I=A\left(h\right)\,{{\mathrm{exp }}}\left(-\frac{t-99}{82}\right)+\left(1-A(h)\right)\,{\mathrm{exp }}\left(-\frac{t-99}{{{\uptau }}}\right)$$

where *h* and τ denote the height of the cell and intracellular decay time, respectively. Here, the unit of height and time are μm and ms, respectively. The first and second terms represent the extracellular and intracellular temporal decay, respectively, which are linearly combined using the contribution ratio *A*(*h*). The time origin of the decay (99 ms) and the extracellular decay time (82 ms) are predetermined by a data fitting of the extracellular MIP phase change by substituting *A*(*h*) = 1. The contribution ratio *A*(*h*) is written as

5 $$A\left(h\right)=\frac{{S}_{{\rm{water}}}}{{S}_{{\rm{water}}}+{S}_{{\rm{cell}}}}$$

where *S*_cell_ and *S*_water_ describe the MIP phase changes, which can be represented by the Lambert-Beer law as

6 $${S}_{{\rm{cell}}}=\mathop{\int }\nolimits_{0}^{h}{{\exp }}\left(\frac{-z}{{D}_{z}}\right){dz}$$

and

7 $${S}_{{\rm{water}}}=\mathop{\int }\nolimits_{h}^{20}{{\exp }}\left(\frac{-z}{{D}_{z}}\right){dz}$$

where *D*_*z*_ is the attenuation length along the z-axis (6.73 μm at 3014 cm^−1^ ^54^), and 20 is the capillary thickness in μm. By substituting Eqs. 6 and 7 into Eq. 5, *A*(*h*) is written as

$$A\left(h\right)=\frac{{{\exp }}\left(\tfrac{-h}{{D}_{z}}\right)-{{\exp }}\left(\tfrac{-20}{{D}_{z}}\right)}{1-{{\exp }}\left(\tfrac{-20}{{D}_{z}}\right)}$$

Here, the spatial distribution of the cell height *h* is estimated by a low-pass filtered phase image, which provides a global feature of a cell, and a literature value of refractive index difference between the inside and outside of a cell (0.0323^55–57^).

The velocity of water molecules passing through the cell membrane can be expressed as

8 $${P}_{d}=1/(\tau (S/V))$$

with the surface area *S* and volume *V* of a cell, which can be calculated from the following equations,

$$S=\sum _{{\rm{all}}\; {\rm{pixels}}}\sqrt{1+\left(\frac{\partial h}{\partial x}\right)^{2}+\left(\frac{\partial h}{\partial y}\right)^{2}}{\Delta x}{\Delta y}$$

and

$$V=\sum _{{\rm{all}}\; {\rm{pixels}}}h(x,y)\Delta x\Delta y$$

respectively, where Δ*x* and Δ*y* are the lengths of a pixel in the x and y directions. The Δ*x* and Δ*y* are 0.44 μm in this study. The resulting values of *S* and *V* are 1404 μm^2^ and 3779 μm^3^, respectively.

### MCR analysis

Prior to MCR analysis, the spatial MIP phase change contrasts reflecting the MIR beam profile are corrected by dividing the MIP phase change images of cells by normalized MIP phase change images of water without cells. Also, the wavenumber-dependent power variation of the MIR light is normalized with the data shown in Fig. 3a. MCR analysis is performed using pyMCR developed by NIST with a non-negativity constrained least-squares regressor. The spectral data with water background subtraction at the nucleolus and lipid droplets and those without water background subtraction outside the cell are used for the initial input spectra in MCR.

We calculate the MIP phase change contributed by each MCR component by the following procedure. MCR decomposes the hyperspectral data into matrices of the concentration distribution ***C*** and the pure spectra ***S*** for each MCR component *i*,

$$H(x,k)={\boldsymbol{CS}}=\sum _{{\boldsymbol{i}}}{{\boldsymbol{C}}}_{{\boldsymbol{i}}}({\boldsymbol{x}}){S}_{i}(k)$$

where *x* and *k* denote the location and the MIR wavenumber, respectively. Each component’s contribution to the MIP phase change at (*x*, *k*) can be calculated as

$${R}_{i}\left(x,k\right)=\frac{{{\boldsymbol{C}}}_{{\boldsymbol{i}}}\left({\boldsymbol{x}}\right){S}_{i}\left(k\right)}{\sum _{{\boldsymbol{i}}}{{\boldsymbol{C}}}_{{\boldsymbol{i}}}\left({\boldsymbol{x}}\right){S}_{i}\left(k\right)}\times \Delta {{\varnothing }}\left(x,\,k\right)$$

where $\Delta \varnothing (x,k)$ is the raw MIP phase change at (*x*, *k*).

## Supplementary information

Supplementary Information

Supplementary Video

## Data Availability

The data provided in the manuscript is available from T.I. upon request.
