# Supplementary material for: Label-free mid-infrared photothermal live-cell imaging beyond video rate
Source: Light Sci Appl. 2023 Jul 19;12:174. doi: 10.1038/s41377-023-01214-2 (PMC10354118; doi:10.1038/s41377-023-01214-2)
Supplement: Supplementary file 1 — Supplementary Information [file 41377_2023_1214_MOESM1_ESM.docx]

**Supplementary Information for**

**Label-free mid-infrared photothermal live-cell imaging beyond video rate**

Genki Ishigane^1+^, Keiichiro Toda^1+^, Miu Tamamitsu^1,2^, Hiroyuki Shimada^2^, Venkata Ramaiah Badarla^2^, and Takuro Ideguchi^1,2,*^

^1^ Department of Physics, The University of Tokyo, Tokyo, Japan

^2^ Institute for Photon Science and Technology, The University of Tokyo, Tokyo, Japan

^+^These authors contributed equally to this work

^*^ Corresponding author: ideguchi@ipst.s.u-tokyo.ac.jp

**Supplementary Note 1: Comparison of parameters of the demonstrated wide-field MIP imaging systems**

Table S1 shows the various parameters of MIR light, visible light, image sensors, and imaging frames of the previously demonstrated wide-field MIP imaging systems and our developed system in this work.

**Table S1 Parameters of previously demonstrated wide-filed MIP imaging systems and our system.**

| Ref.  (Year) | MIR light  Pulse energy (excitation area)  Pulse duration, repetition rate  Wavenumber tuning range | Visible light  Pulse duration Repetition rate | Image sensor  Frame rate  Full-well capacity | Imaging frame rate |
| --- | --- | --- | --- | --- |
| [^1^]  (2019) | Pulsed OPO  ~250 nJ* (~40 μm x 40 μm)  50 ns*, 20 kHz  1,175-1,800 cm^-1^ | LED  914 ns  20 kHz | 2.5 kHz  19 ke^-^ | 2 Hz |
| [^2^]  (2019) | CW QCL  16-40 mW (~40 μm x 60 μm)*  2 ms, 250 Hz  1,450-1,640 cm^-1^ | LED  CW | 10 kHz  ~20 ke^-^* | 10 Hz |
| [^3^]  (2019) | Pulsed OPO  ~110 nJ (~40 μm x 60 μm)*  10 ns, 1 kHz  2,700-3,600 cm^-1^ | SHG of fs laser  900 ns  1 kHz | 100 Hz  30 ke^-^ | 1 Hz |
| [^4^] (2020) | Pulsed QCL  ~100 nJ (~40 μm x 40 μm)*  1 μs, 1 kHz  1,450-1,640 cm^-1^ | SHG of ns laser  ~10 ns  1 kHz | 100 Hz  10 ke^-^* | 0.02 Hz |
| [^5^]  (2020) | CW QCL  500 mW* (~460 μm x 460 μm)  0.83 ms, 600 Hz  900-1,900 cm^-1^ | LED  CW | 500 Hz  ~1 Me^-^ | ~0.1 Hz |
| [^6^] (2021) | Pulsed QCL  ~100 nJ (~30 μm x 70 μm)  1 μs, 1 kHz  1,450-1,640 cm^-1^ | SHG of ns laser  ~10 ns  1 kHz | 20 Hz  10 ke^-^ | ~0.1 Hz* |
| [^7^] (2021) | Pulsed QCL  ~40 nJ* (~70 μm x 70 μm)  1 μs, 200 kHz  1,500-1,700 cm^-1^ | SHG of fs laser  200 ns  200 kHz | 400 Hz | 0.2 Hz |
| [^8^]  (2022) | Quasi-CW QCL  160 mW (~64 μm x 64 μm)  5 µs, 100 kHz burst mode  2,015-2,220 cm^-1^ | LED  CW | 50 kHz | 2.5 Hz |
| This work (2023) | Pulsed OPO  ~10 uJ (~85 μm x 85 μm)  10 ns, 1 kHz  2,800-3,250 cm^-1^ | SHG of ns laser  10 ns  1 kHz | 100 Hz  2 Me^-^ | 50 Hz |

*These values are estimated by the authors from the displayed data and specification sheets of devices because these are not clearly specified in the referenced papers.

The MIP phase changes induced with a pulsed OPO, pulsed QCL, and CW QCL can be deduced from the simulation results depicted in Fig. 1b and the MIR pulse energies shown in Table S1. The MIP phase change can be quantified solely based on the MIR pulse energy for pulse durations that do not lead to saturation. For instance, a pulse duration of < 1 μs satisfies the optimal condition only for observing objects larger than 2 μm. For pulse durations that do not meet this criterion, such as the CW QCL, the MIP phase change must be evaluated by taking into account the saturation effect, as outlined in Fig. 1b.

**Supplementary Note 2: Derivation of the optimal pulse duration and repetition rate of MIR OPO.**

Figure S1 illustrates the equivalent simulation as Fig. 1b for a constant pulse energy instead of a constant peak power, which better represents situations using a ns-OPO. The figure shows that the shorter pulses do not lose the MIP phase change (the quantitative capability, in other words) due to thermal diffusion.


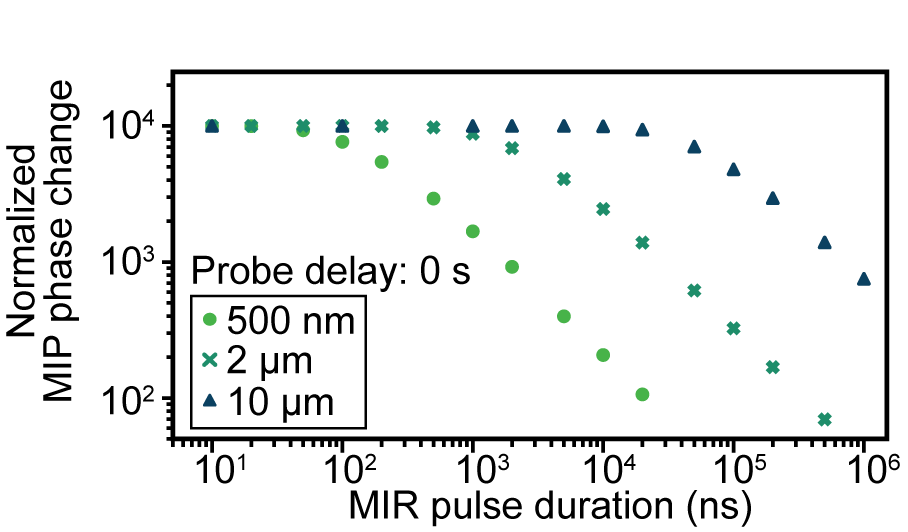


**Figure S1 MIP phase change depending on the MIR pulse duration for constant pulse energy.**

**Supplementary Note 3: Schematic of ns-PPLN-OPO.**

Figure S2 illustrates a schematic of the nanosecond periodically poled lithium niobate optical parametric oscillator (ns-PPLN-OPO). Here, we provide additional information on this laser. The facets of the PPLN crystal are coated for anti-reflection with a reflectance of less than 1.0% at 1,030~1,080 nm and 5,560~7,250 cm^-1^, and less than 5% at 2,220~5,560 cm^-1^. The pump light from the Nd:YAG Q-switched laser is loosely focused onto the crystal by a lens with a focal length of 200 mm. The OPO cavity is designed for resonating the NIR signal pulses, comprised of two ZnSe flat mirrors that are separated by a distance of about 80 mm. The mirrors are coated for high reflectance at 6,060~8,000 cm^-1^, as well as for high transmission at 1,064 nm and 1,670~ 3,330 cm^-1^. The long-pass filter after the resonator passes the idler light at 2,080~3,970 cm^-1^. The maximum velocity of the translation stage for adjusting the poling period of the PPLN crystal to tune the MIR wavenumber is 0.1-1 mm s^-1^, allowing for the acquisition of 40 spectral images within 0.8 s through continuous sweeping. Note that alignment is not necessary after wavelength changes.

**
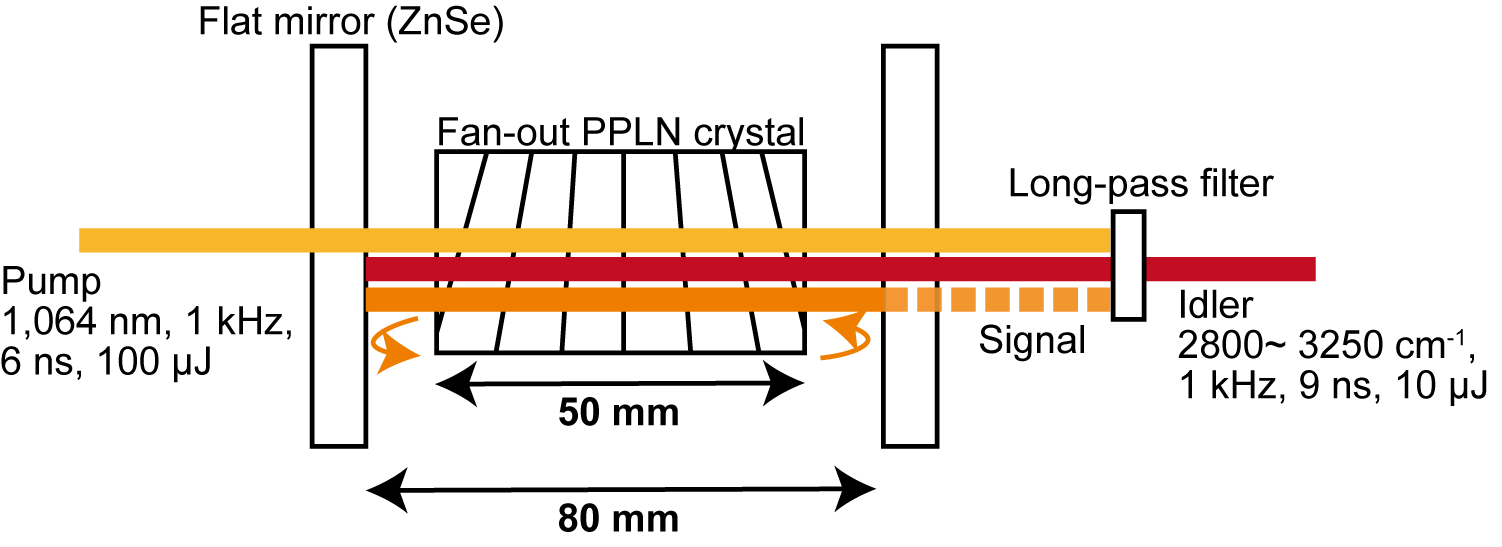
**

**Figure S2 Schematic of ns-PPLN-OPO.**

**Supplementary Note 4: Procedures of phase image reconstruction and visibility evaluation**

Figure S3a is a hologram captured by the image sensor. Figure S3b is the Fourier transform of Fig. S3a, including three spectral components, a non-interferometric term in the center, and two neighboring interferometric terms. Inverse Fourier transform of the cropped area (within the circle of Fig. S3b) provides a complex amplitude image of the electric field, from which the quantitative phase image can be obtained by taking the phase components (see Fig. S3e). Figure S3f shows the visibility $v$ ($= 2\beta/\alpha)$, where $\alpha$ and $\beta$ are the amplitudes of the inverse Fourier transforms of the interferometric and non-interferometric terms (Fig. S3c and 3d), respectively. The number of electrons contributed to the reconstruction of the phase image (=$N_{\text{electron}}$) is calculated from $\beta$ with the sensor’s parameters such as full-well capacity, bit depth, and gain.


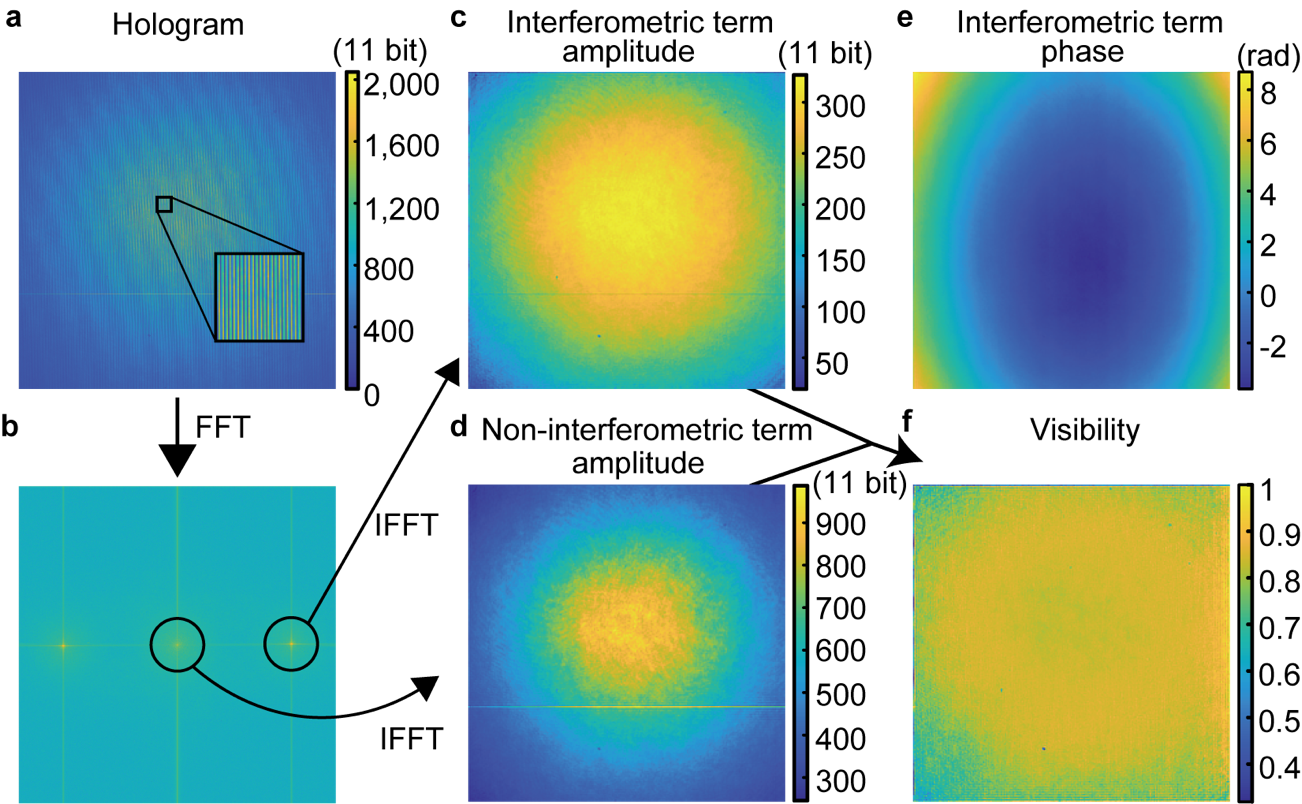


**Figure S3 Procedure to calculate the phase image and the visibility from a measured hologram.** **a** Measured hologram. **b** Fourier transform of **a**. **c, e** Amplitude and phase images derived from inverse Fourier transform of the interferometric term in **b**. **d** Amplitude image derived from inverse Fourier transform of the non-interferometric term in **b**. **f** Visibility calculated from **c** and **d**.

**Supplementary Note 5: Broadband MIP spectro-imaging of a single living cell in D_2_O**

We measure spectra of a single live COS7 cell in D_2_O-based PBS to eliminate the effect of absorption by OH bonds. 40 MIP images are acquired in the range of 2,800~3,250 cm^-1^ (under the same conditions as Fig. 7) and subjected to multivariate analysis. Figures S4a-c, d, e and f show spatial distributions of the three MCR components, a phase image in a MIR-OFF state, a merged image of the three MCR images, and spectra of the three MCR components, respectively.


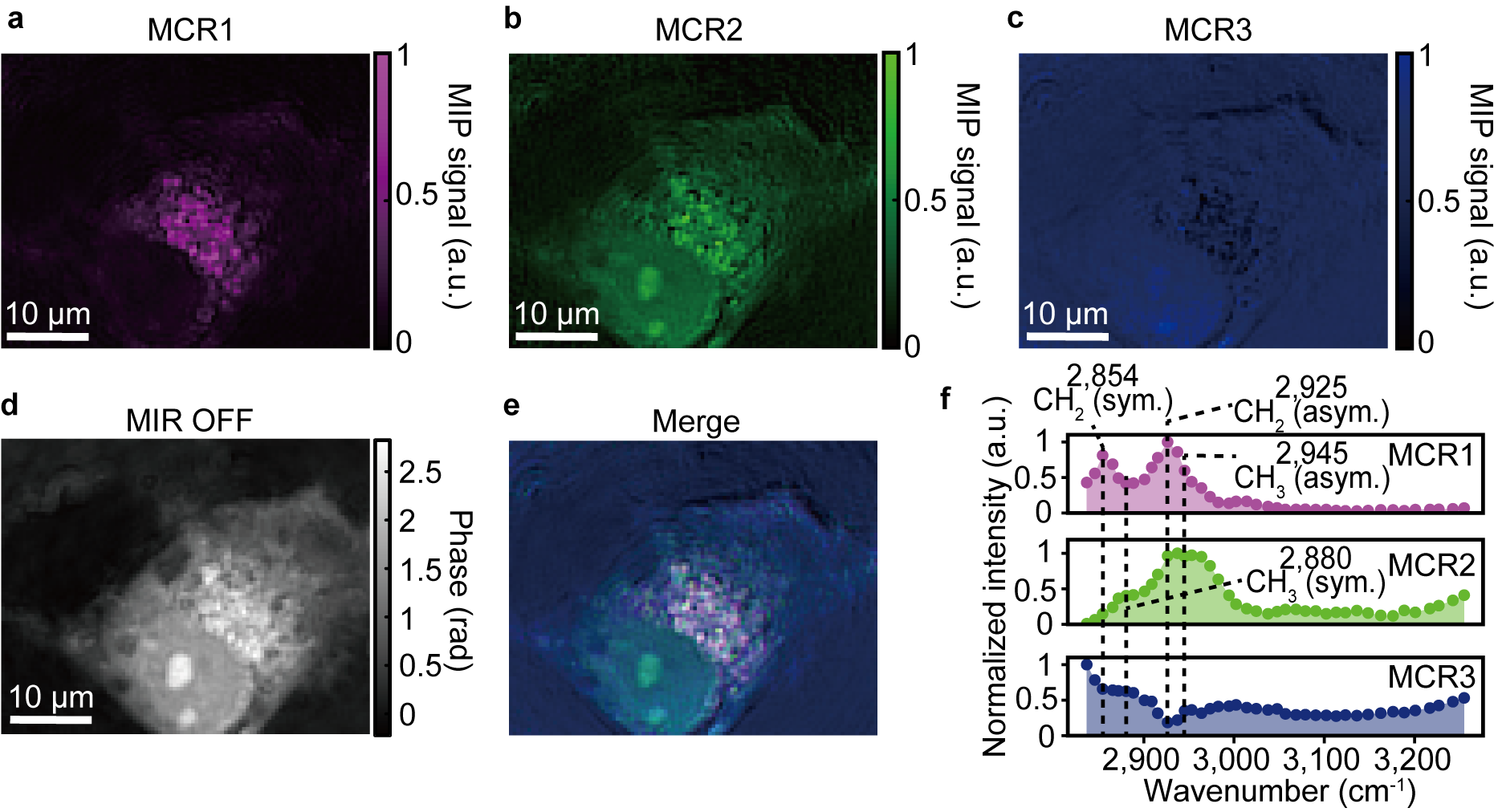


**Figure S4 Multivariate analysis of living COS7 cells in D_2_O-based PBS.** 40 MIP images are acquired in the range of 2,800~3,250 cm^-1^. **a-c** Images of each MCR component. **d** A phase image. **e** A merged image of the three MCR images. **f** MIR spectra of each MCR component. MCR: multivariate curve resolution. sym.: symmetric vibrations. asym.: asymmetric vibrations.

**Reference**

1. Bai, Y. R. *et al*. Ultrafast chemical imaging by widefield photothermal sensing of infrared absorption. *Science Advances* **5**, eaav7127 (2019).
2. Toda, K. *et al*. Molecular contrast on phase-contrast microscope. *Scientific Reports* **9**, 9957 (2019).
3. Zhang, D. L. *et al*. Bond-selective transient phase imaging via sensing of the infrared photothermal effect. *Light: Science & Applications* **8**, 116 (2019).
4. Tamamitsu, M. *et al*. Label-free biochemical quantitative phase imaging with mid-infrared photothermal effect. *Optica* **7**, 359-366 (2020).
5. Schnell, M. *et al*. All-digital histopathology by infrared-optical hybrid microscopy. *Proceedings of the National Academy of Sciences of the United States of America* **117**, 3388-3396 (2020).
6. Toda, K., Tamamitsu, M. & Ideguchi, T. Adaptive dynamic range shift (ADRIFT) quantitative phase imaging. *Light: Science & Applications* **10**, 1 (2021).
7. Zong, H. N. *et al*. Background-suppressed high-throughput mid-infrared photothermal microscopy via pupil engineering. *ACS Photonics* **8**, 3323-3336 (2021).
8. Paiva, E. M. & Schmidt, F. M. Ultrafast widefield mid-infrared photothermal heterodyne imaging. *Analytical Chemistry* **94**, 14242-14250 (2022).
